# QASI: A collaboration for implementation of an independent quality assessment programme in India

**DOI:** 10.4102/ajlm.v5i2.442

**Published:** 2016-10-12

**Authors:** Adrienne F.A. Meyers, Michèle Bergeron, Madhuri Thakar, Tao Ding, Alexandre Martel, Paul Sandstrom, Bharati Mahajan, Philip Abraham, Sandhya Kabra, Namita Singh, Trevor Peter, Terry B. Ball

**Affiliations:** 1 National HIV & Retrovirology Laboratories, Public Health Agency of Canada, JC Wilt Infectious Diseases Research Centre, Winnipeg, Canada; 2 Department of Medical Microbiology and Infectious Diseases, University of Manitoba, Winnipeg, Canada; 3 Department of Medical Microbiology, University of Nairobi, Kenya; 4 National AIDS Research Institute, Pune, India; 5 National AIDS Control Organization, New Delhi, India; 6 Clinton Foundation’s HIV/AIDS Initiative, Botswana; 7 Department of Immunology, University of Manitoba, Winnipeg, Canada

## Abstract

**Objective:**

The HIV pandemic remains a significant global health concern. Accurate determination of CD4+ T-cells in patient samples relies on reliable CD4 enumeration. The Quality Assessment and Standardization programme for Immunological measures relevant to HIV/AIDS (QASI) programme of the Public Health Agency of Canada provides clinical laboratories from resource-limited countries with a mechanism to evaluate the quality of CD4 testing and develop the implementation of an independent national External Quality Assessment (EQA) programme. This study describes how QASI helped develop the capacity for managing a sustainable national CD4 EQA programme in India.

**Design:**

Supported by the Public Health Agency of Canada and Clinton Foundation HIV/AIDS Initiative, QASI engaged with the National AIDS Control Organization and the Indian National AIDS Research Institute to assist in technology transfer in preparation for the implementation/management of an independent CD4 EQA programme. Technology transfer training was provided to support corrective actions and to improve the quality of CD4 testing. Inter-laboratory variation of EQA surveys between pre- and post-skill development was compared.

**Results:**

Prior to training, coefficient of variation values were 14.7% (mid-level CD4 count controls) and 39.0% (low-level). Following training, variation was reduced to 10.3% for mid-level controls and 20.0% for low-level controls.

**Conclusion:**

This training assisted the National AIDS Control Organization and the Indian National AIDS Research Institute in identifying the information necessary for management of an EQA programme, and developed the foundation for India to provide corrective actions for sites with challenges in achieving reliable results for CD4 enumeration. This led to a demonstrable improvement in CD4 testing quality and illustrates how country-specific training significantly improved CD4 enumeration performance for better clinical management of HIV care in India.

## Introduction

Infection by HIV and progression to death in the form of AIDS is responsible for significant morbidity and mortality worldwide, despite considerable efforts to prevent and/or treat the disease. Recent figures from the World Health Organization estimate that 36.9 million people worldwide are living with HIV and more than two-thirds of these individuals are in resource-limited settings.^1^ While this number is an increase over the 2010 figures of 33.3 million, it represents the fact that more people are able to access and receive antiretroviral therapy (ART) for their infections. According to UNAIDS, 15.8 million people worldwide are accessing ART.^2^

Immune responses to HIV infection are largely mediated by CD4 T-lymphocytes – depletion of which reflects disease progression and AIDS.^3,4,5^ While the latest World Health Organization Guidelines recommend treatment for all infected with HIV, irrespective of CD4 counts, many resource-limited countries have yet to adopt these recommendations due to constraints, and CD4 enumeration remains the hallmark for staging and monitoring patients on ART.^6^ New targeted efforts aimed at the HIV epidemic are focused on the 90-90-90 initiative proposed by UNAIDS that by 2020, 90% of individuals living with HIV will know their status, 90% of those infected with HIV will have access to ongoing ART and 90% of those on ART will achieve suppression of HIV infection.^7^ As such, it is critical to properly and rapidly detect HIV infection and responses to therapy. Reliable CD4 measurements are critical to clinical decision-making in situations where viral load data is unavailable.^8,9^

The most common technology utilised for CD4 T-cell enumeration is flow cytometry. The complexity of this technology and the lack of a CD4 gold standard against which to measure accuracy can produce misleading results that affect medical decisions in resource-limited areas where infrastructure and levels of training required can be a challenge.^10,11^

To achieve accuracy and reproducibility of laboratory based testing, both internal and external quality control measures are essential.^8,9,12^ Since 1996, the National Laboratory for HIV Immunology from the Public Health Agency of Canada has been providing the Quality Assessment and Standardization programme for Immunological measures relevant to HIV/AIDS (QASI) in an effort to assist resource-limited regions with CD4 testing.^13^ Currently over 1400 laboratories from more than 50 countries are enrolled and actively participating in QASI.

In addition to the provision of an external quality assessment (EQA) programme for relative and absolute CD4 enumeration, QASI also encompasses several elements that constitute a comprehensive quality management system. QASI has developed a framework to train and support implementation of sustainable quality assessment programmes in resource-limited countries. QASI provides free quality control panels; individual, national and global performance assessments; and supports capacity building activities toward quality improvement such as remedial action plans, biotechnology transfer workshops based on the ‘training-of-trainers’ model, and web-based platforms to assist with quality management activities at a national level.

In 2005, the National AIDS Control Organization (NACO) of the Government of India joined QASI to help initiate an external quality assessment programme for CD4 count enumeration in India, with support from the Clinton Health Access Initiative. The Indian National AIDS Research Institute (NARI) in Pune, India, being the apex laboratory in India, acted as a coordinator for the distribution of the QASI proficiency panel to the enrolled laboratories of the National HIV/AIDS programme. In September 2009, NACO, NARI and the Clinton Foundation engaged with QASI on technology transfer so that India could implement its own national CD4 EQA programme. A technology-transfer workshop was conducted by QASI to NARI. Additionally, NACO and NARI have also initiated focused training workshops for the CD4 testing laboratories enrolled as participants.

This report describes how tailored and concerted intervention strategies can significantly improve laboratory performance at the national level, highlights the critical elements covered during training in preparation for launch of the Indian National EQA Program for CD4 enumeration, and provides insight to lessons learned during the process.

## Design and methods

CD4 EQA was initiated in India in 2005 with support from the Clinton Health Access Initiative. Three times a year, NARI distributed proficiency panels provided by QASI, consisting of two specimens, to all 219 enrolled CD4 laboratories of the Indian National HIV/AIDS programme. The CD4 testing centres processed the samples as per their established procedures and reported the results back to QASI, with a copy to NARI. QASI analysed the performance of all the laboratories and reported back to NARI the individual, national, and global performance reports, as well as a set of recommendations for remedial action and follow-up where required.

To ensure the sustainability and ongoing impact of this initiative, NACO/NARI engaged with QASI to assist in a series of technical consultations and discussions, culminating in a training-of-trainers workshop held in 2009. The goal of these meetings was for QASI to assist NARI with planning procedures so that India could implement and manage an independent national CD4 EQA programme.

### Technology transfer training

#### QASI CD4 enumeration workshop: September 2009

The QASI CD4 technology transfer workshop was conducted in September 2009 at the NARI in Pune, India. Six people from NARI, including the Laboratory-in-Charges, technical and quality managers, and statisticians, participated in the workshop. Additionally, three Laboratory-in-Charges from the National Reference Laboratory participated in the workshop. Workshop objectives included skills transfer related to specific instruments, logistics of panel selection and procurement, policy development (EQA management team, participants, enrolment, standard operating procedures, panel testing, shipment, reporting, remedial action response), capacity building (planning and development of budget, identification of financial support), and training on the technical capacity, operation and management of the QASI Web-based software application. QASI has the capacity to offer instrument-specific training for all CD4 technologies in use. The workshop was designed with this in mind so that all instruments in use throughout the country were included in the training modules, thus allowing the team to develop specific skills to evaluate and assist performance by participants in a precise and specialised manner. This training was also critical to the adaptation of the QASI EQA software to the India-specific management of the EQA programme, so as to enable an independent national CD4 EQA programme for NACO’s HIV care and treatment programme.

The design of the technology transfer workshop was established by first investigating potential sources of variation in CD4 enumeration within the country by means of examination of EQA results for trends and bias, followed by a comparison of national performance with global performance data. The workshop agenda was developed and approved based on country-specific details, including instruments used by participants, mechanisms of communication with participants, and modes of transportation for distribution of panels/collection of data. The training modules, which addressed internal and external quality control activities, included practical sessions targeting troubleshooting and quality management using a dedicated web application. In total, nine people with advanced instrument-specific knowledge were trained through problem-solving exercises and quality-management simulation activities using the QASI web platform.

#### NACO- and NARI-designed laboratory technology training: November–December 2009

To assist in corrective actions and to improve the quality of CD4 testing centres, focused training sessions for individual CD4 testing laboratories were initiated by NARI and NACO in April 2009, which were continued after the technology transfer workshop delivered by QASI. Eight training sessions were conducted with the collaboration of instrument manufacturers for more than 100 CD4 testing laboratories. A total of 100 technicians from the testing centres participated in the training. The agenda was tailor-made for FACSCalibur™, FACSCount™ and Cyflow^®^ Counter users, who represented more than 98% of the enrolled CD4 testing laboratories in India. The workshop included hands-on equipment training, troubleshooting procedures and pipette calibration. The training module also included internal quality control procedures, information about participation in EQA and response to remedial action in the event of unsatisfactory performance. Two additional sessions were conducted for the FACSCalibur users, specifically to address gating issues related to MultiSet™ analysis of EQA-stabilised blood samples.

### Evaluation of performance pre-/post-initiation of training of technologists

To study the impact of comprehensive training on CD4 enumeration abilities, national performance was evaluated based on the results generated from six consecutive participations in QASI surveys (QASI surveys 19 [October 2008] to survey 24 [June 2010]). Each survey provided two controls of commercially-stabilised whole blood product for CD4 testing: low-level (100–150 cells/µL) and mid-level (450–700 cells/µL). The first three sessions took place prior to initiation of the technologist training, and the latter three took place after the training of all technologists from 100 laboratories was completed. National performance was evaluated against the global performance of QASI participants for each session examined.

## Results

### Pre-technology and targeted training transfer

**FIGURE 1:**
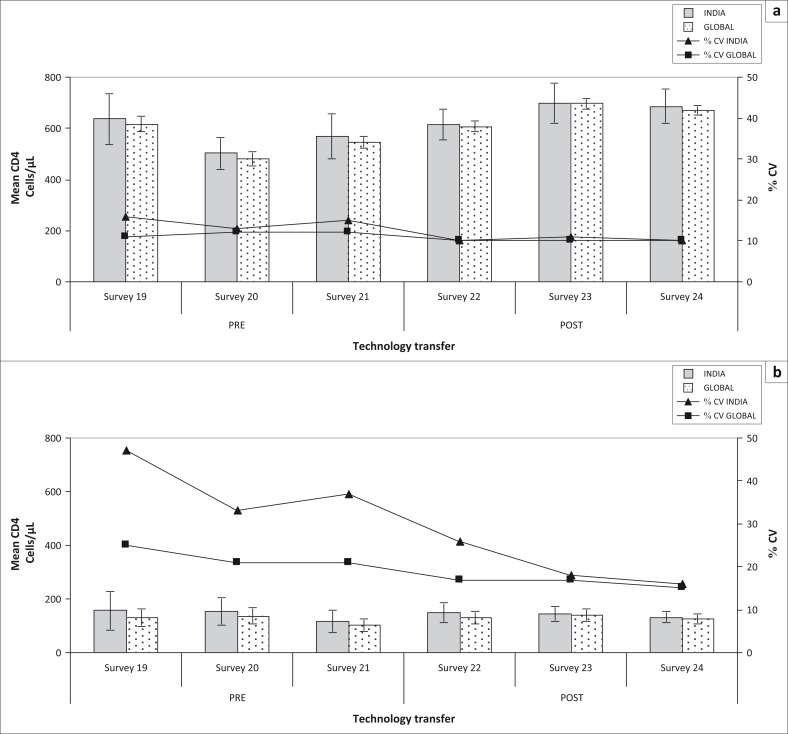
Pre- vs post-training national and global CD4 absolute count performance. Comparison of national and global performance across six consecutive surveys, reflecting the impact of technology transfer provided by both QASI and NARI between surveys 21 and 22. Pre-technology transfer reflects QASI sessions 19 through 21 (October 2008, February 2009, June 2009). QASI advanced workshop training was held in September 2009 at NARI in Pune, India. Post-technology transfer reflects QASI sessions 22 through 24 (October 2009, February 2010, June 2010). Group mean CD4 count, standard deviation (error bars) and %CV are illustrated for mid-level (a) and low-level (b) CD4 count controls. Note the drop in %CV for India from 14.7% to 10.3% (a) and from 39.0% to 20.0% (b).

**FIGURE 2:**
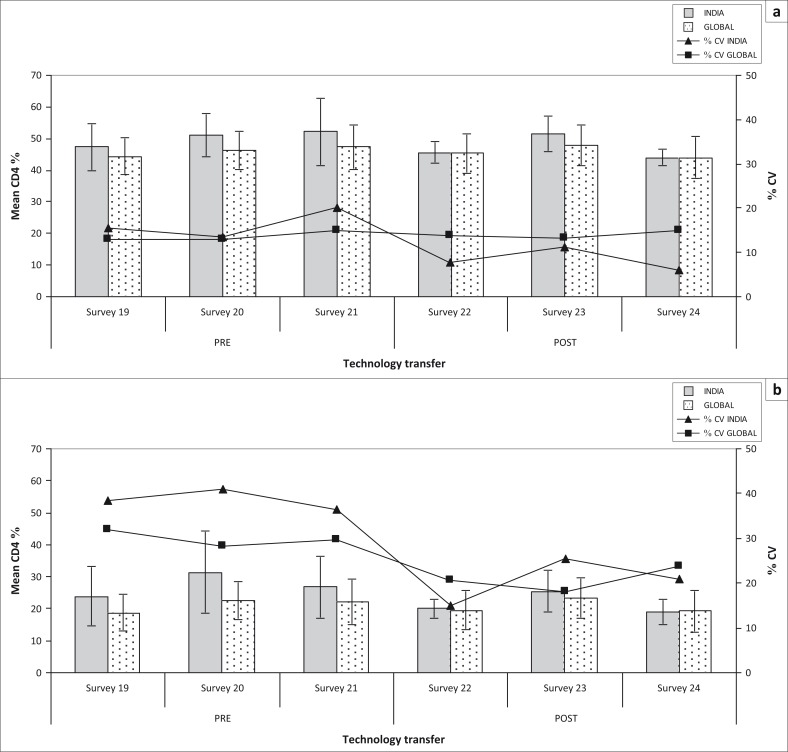
Pre- vs post-transfer national and global CD4 percentage performance. Comparison of national and global performance across six consecutive surveys reflecting the impact of technology transfer provided by both QASI and NARI between surveys 21 and 22. Pre-technology transfer reflects QASI sessions 19 through 21 (October 2008, February 2009, June 2009). QASI advanced workshop training was held in September 2009 at NARI in Pune, India. Post-technology transfer reflects QASI sessions 22 through 24 (October 2009, February 2010, June 2010). Group mean CD4 lymphocyte percentages, standard deviation (error bars) and %CV are illustrated for mid-level (a) and low-level (b) CD4 count controls. Note the drop in %CV for India from 16.4% to 8.3% (a) and from 38.6% to 20.4% (b).

Data from QASI surveys 19 to 21 (October 2008, February 2009 and June 2009) were used to assess pre-workshop and targeted training performance in India. In that period, more than 50 laboratories in India reporting absolute counts participated in QASI. The main CD4 technologies used were FACSCalibur (*n* = 25; 22%), FACSCount (*n* = 48; 43%) and Cyflow Counter (*n* = 39; 35%). As depicted in Table 1, not all instruments were available for use in the QASI sessions for both pre- and post-technology training. Reasons for this involved issues with the instruments (i.e., breakdown in machine function). Initial observations of the submitted data showed that the inter-laboratory coefficient of variation (%CV) for both relative and absolute CD4 measurements in India was higher than values obtained by the global performance, especially with low-level CD4 count controls (Figures 1 and 2). Further analysis identified acute problems with specific technologies. The largest %CV levels of variation were identified by participants using the FACSCalibur, with 25.6% for mid-level CD4 count controls and 55.4% for low-level controls. Cyflow Counter measurements elicited 14.4% for mid-level controls and 26.3% variation for low-level controls. The best performance was achieved among the FACSCount users, who had %CV that were less than 8.5% for both levels of CD4 control samples. These data suggested the focus should be on providing remedial action for users of FACSCalibur and Cyflow Counter, due to the higher levels of variation with these two technologies in comparison with the FACSCount performance (Table 1).

**TABLE 1:** Performance of specific CD4 technologies in India for low- and mid-level CD4 count controls at pre- and post-technology transfer.

Parameters	Global [*n*]	India				
	Mean ± SD cells/µL	CD4 technology	Mean cells/µL	s.d.	%CV	*n*
**PRE technology transfer (Survey 21, June 2009)**						
Low CD4 count	103 ± 22.0 [463]	FACSCalibur	148.9	82.5	55.4	25
		Cyflow Counter	117.9	31.0	26.3	22
		FACSCount	96.9	7.9	8.2	24
**POST technology transfer (Survey 24, June 2010)**						
Low CD4 count	125 ± 19.0 [537]	FACSCalibur	121.4	27.1	22.4	25
		Cyflow Counter	141.6	17.6	12.4	39
		FACSCount	127.4	15.0	11.8	47
**PRE technology transfer (Survey 21, June 2009)**						
Mid CD4 count	546 ± 62.9 [461]	FACSCalibur	648.6	166.2	25.6	25
		Cyflow Counter	573.8	82.6	14.4	22
		FACSCount	537.3	44.5	8.3	23
**POST technology transfer (Survey 24, June 2010)**						
Mid CD4 count	669 ± 68.6 [539]	FACSCalibur	660.0	88.1	13.4	25
		Cyflow Counter	671.3	56.0	8.4	39
		FACSCount	695.8	55.0	7.9	48

*Source:* QASI Workshop in September 2009 at NARI in Pune, India

s.d., Standard Deviation; *n*, number of participants.

In examining protocols utilised by sites equipped with FACSCalibur instruments, it was determined they were primarily relying on the use of the automated attractor MultiSet software (BD BioSciences). In QASI’s experience, the attractor algorithm associated with the software can have a limited capacity to adapt to some stabilised whole blood products provided as EQA quality control panels.^11,14,15^ Fluorescence intensities are significantly different in these specimens as compared with fresh whole blood samples. This characteristic is not, however, exclusive to stabilised controls, as patient samples may behave similarly due to drug therapy or exposure to environmentally hostile conditions during transit. Both NARI and QASI dedicated special attention to FACSCalibur users during the workshop and in the follow-up training period, emphasising the use of the manual analysis mode, where attractor regions are required to be readjusted in order to achieve accurate values. With respect to Cyflow Counter users, several laboratories were using a single CD4 antibody on a single-parameter histogram. Training raised the importance of changing the cursor position of the gating strategy in order to accurately identify CD4 T-cell expression and reduce monocyte contaminants. The option of utilising a CD4 x Side Scatter heterogeneous algorithm was also introduced. These issues were then emphasised in the subsequent training workshops organised for the CD4 testing laboratory participants.

### Post-technology and targeted training transfer

Post-technology transfer data analysis was based on QASI surveys 22 to 24 (October 2009, February 2010, June 2010). The analysis demonstrated that the %CV for both CD4 percentages and CD4 absolute counts improved following the training programme. The average %CV of reported CD4 percentages before the completion of all training workshops was nearly twice the average %CV of results obtained in surveys following the training; 16.4% versus 8.3% for the mid-level CD4 control and 38.6% versus 20.4% for the low-level control (Figure 2). As inter-laboratory variation improved dramatically, so did accuracy, closing the gap between the global mean value and the national mean value.

The same trend was observed with absolute CD4 measurements, with an important reduction in the %CV values following workshop training (Figure 1). Prior to training, mean %CV values were 14.7% for the mid-level CD4 controls and 39.0% for the low-level controls. Following training, variation was reduced to 10.3% for mid-level and 20.0% for low-level controls.

BOX 1 Lessons learned.

Targeted, instrument-specific training reduces inter-laboratory variation and improves the quality of CD4 enumeration.

Country-specific workshops tailored to specific needs and with a focus on sample procurement/preparation, logistics and corrective action aid in the preparation for a successful implementation and management of independent EQA programmes for CD4 enumeration.

Thus, both FACSCalibur and Cyflow Counter users showed dramatic improvement in their performance following the workshop and subsequent training. The combined training provided by both NARI and QASI was successful in addressing country- and laboratory-specific issues, resulting in quality improvements in CD4 enumeration methodologies used by more than half of the participating Indian laboratories and thus increasing their ability to provide better clinical and prognostic data for the management of HIV-infected patients.

A dedicated web application was developed as an extension of the web platform application utilised by QASI for India in order to assist NARI with pilot trials to pave the way for the eventual launch of their first independent national programme. In preparation for the launch, NARI conducted six pilot surveys while still participating in QASI. This transitional phase allowed the Indian EQA team to familiarise themselves with software functions, while also assessing and optimising all aspects of the logistics, such as selection and acquisition of quality control panels, identification and distribution of transport tubes, and policy development on subjects such as reporting and remedial action, so as to ensure a smooth transition during National EQA implementation.

## Conclusion

QASI has more than 20 years of experience in assisting the implementation of activities for quality improvement and best practice for quality assurance in developing countries and, as a part of its overall objective, QASI is dedicated to capacity building and training for countries wishing to develop and implement their own national or regional quality assessment programmes for CD4 enumeration. This report illustrates that specifically tailored workshops designed by QASI, and follow-up training provided by NACO and NARI, led to increased CD4 testing accuracy and provided a foundation for the development and implementation of an independent national quality assessment programme led by NARI and NACO in India. NACO and NARI launched the Indian CD4 national programme in June 2011, and it has been operating independently since then.

The importance of targeted training to diminish inter- and intra-laboratory variation in cell enumeration is well-established and demonstrates that training works. The joint efforts of QASI, NACO, NARI and the Clinton Health Access Initiative in conducting the technology transfer and specific training were extremely useful in improving laboratory performance and providing reliable results and, presumably, better and more effective patient care. In addition, the workshop training provided led to the development of a local EQA management team for the implementation of an independent national EQA programme for CD4 enumeration. The QASI workshop identified key areas to be considered for programme development, including mechanisms for financial sustainability, selection of an appropriate source material for proficiency panels, technical expertise in website management, data collection and analysis, reporting methods, and policies for provision of remedial action for those participants with unsatisfactory results.

This report highlights a phased approach to launching independent EQA programmes for CD4 enumeration, which evolves from initial participation in an EQA programme to gradual stages of increased responsibility in providing services to a country or region until the eventual launch of a fully functional, independent national/regional programme. This model that QASI utilises includes tailored, country-specific training workshops that highlight the strengths and challenges a specific country faces as they prepare for programme development. With the gradual transition to independence, countries are able to properly develop methods and policies that will allow for sustainable programme provision to participants and develop their own unique training modules specific for participants in the region. In addition, the training and feedback provides early detection of systematic problems associated with reagents, instruments, or operations, provides objective evidence of testing quality, indicates areas that need improvement, and identifies ongoing training needs. This method helps to better identify the resources and/or needs for developing laboratory quality standards and EQAs, and guides the organisations in providing support where significant gaps are identified. Ultimately, the ability of a country to develop their own National EQA programme for HIV treatment and care is tightly linked to both improved diagnostics and clinical management of patients.

Our experience demonstrates the impact of effective interventions and the importance of collaboration and commitment of a national reference laboratory and partners to improving healthcare systems in a country.

In conclusion, the data confirm that participation in an EQA programme results in better diagnostic accuracy, and that technology transfer around EQA programmes can be transferred reproducibly.
